# Effectiveness of Silver Diamine Fluoride for Preventing Occlusal Caries in the Primary Teeth of Preschool Children: Protocol for a Randomized Controlled Trial

**DOI:** 10.2196/35145

**Published:** 2022-05-23

**Authors:** Duangporn Duangthip, Shuyang He, Sherry Shiqian Gao, Chun Hung Chu, Edward Chin Man Lo

**Affiliations:** 1 Restorative Dental Sciences Faculty of Dentistry The University of Hong Kong Hong Kong China; 2 Applied Oral Sciences & Community Dental Care Faculty of Dentistry The University of Hong Kong Hong Kong China; 3 Department of Stomatology School of Medicine Xiamen University Xiamen China

**Keywords:** silver diamine fluoride, sodium fluoride, children, early childhood caries, prevention

## Abstract

**Background:**

Tooth decay is a significant public health problem globally. The caries-arrest effectiveness of 38% silver diamine fluoride (SDF) has been well documented. However, information on the caries-preventive effect of SDF on primary teeth is insufficient.

**Objective:**

The aim of this trial is to investigate the effectiveness of semiannual application of 38% SDF and that of 5% sodium fluoride (NaF) varnish when compared with placebo control for preventing occlusal caries in the primary molars of preschool children over 30 months.

**Methods:**

This 3-arm, parallel design, double-blind, randomized controlled trial involves 791 preschool children. Children are randomly allocated to receive 1 of 3 interventions as follows: Group 1, 38% SDF; Group 2, 5% NaF varnish; and Group 3, placebo control (tonic water). The intervention and dental examination will be carried out every 6 months. A parent-administered questionnaire, including the children’s demographic background and oral health–related behaviors, has been collected at baseline. Follow-up examinations to detect new caries development will be conducted every 6 months by a masked examiner. Caries development will be diagnosed at the cavitation level. Chi-square tests and logistic regression analyses will be adopted. A 2-level logistic regression analysis will be performed to investigate the effects of the study interventions and other potential confounding factors on the development of occlusal caries.

**Results:**

This study was started on September 1, 2020, and the recruitment process ended on September 30, 2021. At present, a total of 791 children are participating in the study. This 30-month clinical trial is expected to be completed in March 2024.

**Conclusions:**

If SDF application is more effective than NaF varnish for preventing caries on occlusal surfaces of primary teeth, it can be a preferred choice for caries prevention in a kindergarten-based program. Results of this trial will provide valuable clinical evidence for the development of oral health strategies and policies on the promotion of child oral health.

**Trial Registration:**

HKU Clinical Registry HKUCTR-2844, https://tinyurl.com/bdhz9yuk; ClinicalTrials.gov NCT05084001, https://clinicaltrials.gov/ct2/show/NCT05084001

**International Registered Report Identifier (IRRID):**

DERR1-10.2196/35145

## Introduction

### Background

Tooth decay or dental caries is a silent epidemic. According to the Global Burden of Disease Study, dental caries in primary teeth remains a public health problem affecting more than 530 million children globally [1]. The term “early childhood caries” (ECC) has been used to describe the presence of one or more decayed, restored, or missing primary teeth in a child younger than 6 years [2]. In Hong Kong, more than half (55%) of 5-year-old children have untreated ECC, and the majority (>90%) of the decayed teeth are left untreated [3]. Children with untreated ECC may experience toothache, oral pain, and infection, leading to poor quality of life [4]. This also affects their family members’ quality of life and eventually impacts the community. Severe consequences of ECC include emergency room visits and hospitalizations with high costs of treatment, and missing school, as well as negative impacts on the ability to learn [2]. In extreme cases, untreated ECC can lead to life-threatening conditions and even death [5].

An epidemiological study found that most (78%) cases of ECC in preschool children in Mainland China involved the primary molars [6]. In Hong Kong, decayed primary molars constituted more than half (56%) of all decayed primary teeth in preschool children [7]. High prevalence of premature loss of primary molars due to untreated tooth decay has been reported [8]. Tooth decay is commonly found on the occlusal tooth surface owing to the complex anatomy of the fissure system, which makes the surface difficult to clean, thereby promoting the accumulation of a bacterial biofilm and increasing the risk of developing carious lesions [6]. In addition, young children are not able to brush their posterior teeth properly, and they may not have supervised toothbrushing during the preschool period. Hong Kong preschool children’s oral health has not improved over the last 2 decades [9]. An epidemiological dental survey in Hong Kong found that the prevalence of caries was much higher in older preschool children (38% at age 3 years and 55% at age 5 years) [3]. Currently, there is no government-subsidized dental care service for preschool children in Hong Kong.

Several strategies, including placement of dental sealant and topical application of sodium fluoride (NaF) varnish, have been proposed to prevent occlusal surface caries. Nevertheless, it is a challenge to place dental sealants in the primary molars of young children because the application is technique sensitive and requires optimum tooth isolation. NaF varnish is another potential agent for preventing dental caries, but it is not cost-effective in the context of government-funded services [10]. The relatively high cost of professionally administered fluoride varnish has hindered its widespread adoption in many countries [11]. Recently, silver diamine fluoride (SDF) has been considered a therapeutic agent for treating cavitated dental caries lesions in young children and those with special needs [12]. Several laboratory studies have documented its antibacterial effects in inhibiting cariogenic bacteria and its remineralizing effects [13]. Results of randomized clinical trials have shown its caries-arresting effects in preschool children [14-16]. No major adverse effects of SDF were observed, except dark staining on treated lesions after application [17]. A systematic review concluded that the caries arrest rate of 38% SDF was as high as 81% (95% CI 68%-89%; *P*<.001) [18]. In light of this, the American Academy of Pediatric Dentistry developed a guideline on the use of 38% SDF for arresting cavitated dentin caries lesions in primary teeth among children [12]. Recently, another systematic review and network meta-analysis concluded that semiannual application of 38% SDF solution showed the highest effectiveness for caries arrest in advanced cavitated lesions on coronal surfaces [19].

Nevertheless, clinical evidence for the dental caries–preventive effect of SDF is scarce [20]. Therefore, to fill this knowledge gap and to provide the needed clinical evidence to guide dental practice and public dental care service, this study aims to investigate the preventive effect of SDF versus NaF varnish and a placebo control on the occlusal surfaces of the primary molars of preschool children. In addition, this study will evaluate parental satisfaction with their child’s dental status and dental appearance, and the adverse effects of SDF application.

### Objectives

The objective of this study is to compare the effectiveness of 38% SDF, 5% NaF varnish, and a placebo control for preventing dentine caries in the primary molars of preschool children when applied semiannually over 30 months.

### Hypothesis

The null hypothesis tested is that there is no difference in the effectiveness of 38% SDF, 5% NaF varnish, and a placebo control for preventing dentine caries of the occlusal surfaces of the primary molars in preschool children when applied semiannually over 30 months.

## Methods

### Ethics Considerations

Ethics approval has been obtained from the Institutional Review Board of The University of Hong Kong/Hospital Authority Hong Kong West Cluster (HKU/HAHKWIRB; IRB reference number: UW20-028).

### Trial Design

This is a 3-arm, parallel-design, double-blind, randomized controlled trial consisting of semiannual application of SDF, semiannual application of NaF varnish, and a placebo control. Similar school-based oral health education will be provided to the children and parents in all participating kindergartens. This study will be reported following the SPIRIT (Standard Protocol Items: Recommendations for Interventional Trials) guidelines (Multimedia Appendix 1). Before subject recruitment, the study was registered on January 17, 2020, at HKU Clinical Registry, which is a publicly accessible database, with the reference number HKUCTR-2844. Later, it was registered as a clinical trial by the United States National Library of Medicine (ClinicalTrials.gov) on October 19, 2021, with the registration number NCT05084001, as an additional registration.

### Patient and Public Involvement

Kindergartens that have participated in the school oral health program provided by the Faculty of Dentistry, The University of Hong Kong were invited. Research assistants will assist in all stages of the trial, mainly as contact persons and as persons in charge of inviting kindergartens, explaining the whole trial to kindergartens, and clarifying their queries. After the principals agreed to take part in this study, an information sheet containing the aim of the study and the details of the research study and procedures was distributed to parents or guardians of the children. Parental consent in written form was obtained before conducting this trial. The participant children and their parents are at liberty to withdraw from this study at any time. They are also free to seek further dental treatment with their own means and preferences.

### Eligibility Criteria

The eligibility criteria for participants are as follows: (1) age 3-4 years, (2) healthy status, (3) presence of at least one caries-free (no cavitation) primary molar, and (4) parental written consent. The exclusion criteria are as follows: (1) no cooperation or refusal to undergo examination, (2) major systemic illnesses, (3) presence of acute pain, infections, gingival ulceration, or stomatitis, (4) known sensitivity to silver or other heavy-metal ions, and (5) current involvement in any other research that may impact this study. At the tooth level, primary molars with developmental defects; presence of dental sealants or fillings; presence of pain, fistulas, abscesses, mobility, or discoloration; or any sign of nonvitality are excluded.

### Recruitment and Examination

All phases of this trial will be carried out in the kindergarten that the children attend. The research team includes dentists and research assistants. Before conducting the trial, the whole team was trained by experienced specialists in dental public health. A calibrated and trained examiner screened the children who had written parental consent. All children were clinically examined, and those who met the inclusion criteria were selected. No dental radiograph examination was conducted. If a child has more than one caries-free primary molar, all of the molars will be included. At baseline and the semiannual follow-up examinations, clinical examination of the children will be conducted by a trained dentist who will be masked to the group assignment of the participant children. All clinical investigations will be conducted in kindergartens. The study children will lie on a bench and will be examined in the supine position. Tooth status will be assessed by careful visual inspection with a dental mirror attached to a handle with a light-emitting diode for intraoral illumination (MirrorLite, Kudos Crown Limited). A microbrush will be used to remove food debris and dental plaque that obstruct inspection. A World Health Organization CPI probe with a 0.5-mm ball tip will be used to confirm the presence of a carious cavity when necessary. Great care will be taken to avoid damaging the tooth surface during probing. The intervention will be free, and participants or kindergartens will not pay any cost for the research. After the examination, parents will receive a brief report on their children’s dental caries status.

Caries will be diagnosed at the cavitation level following the criteria recommended by the World Health Organization. Drying of the tooth surface with air blow will not be carried out in this study. An occlusal tooth surface will be recorded in one of the following categories: (1) sound, (2) caries confined to the enamel or into dentin but without cavitation, (3) cavitated dentin caries (active), (4) cavitated dentin caries (arrested), (5) filled surface, (6) extracted tooth, and (7) tooth with nonvital signs, such as abscess or fistula. The decayed, missing, and filled tooth surface (dmfs) index will be adopted for documenting dental caries experience. Oral hygiene status will be measured using the visible plaque index (VPI). The buccal and lingual surfaces of 6 index teeth (Fédération Dentaire Internationale tooth numbers 55, 51, 63, 71, 75, and 83) will be thoroughly examined. The presence or absence of visible plaque on the occlusal surface will be recorded. Black staining on each surface will be clinically observed and recorded (yes/no). A random sample of 10% of the study children will be re-examined on the same day at the baseline and follow-up examinations to evaluate intraexaminer reproducibility. The study follow-up is every 6 months. If some parents of the study children seek early professional intervention, all the sealed, restored, or extracted teeth will be recorded as study treatment failure.

### Questionnaire Survey

A parental questionnaire consisting of 3 fields (child’s information, child’s oral health–related behavior, and family information) will be administered at baseline and at the 30-month follow-up visit. It will collect information regarding the child’s oral hygiene practice, use of fluoride agents, dental visit behavior, snacking habits, parental educational level, and family income. The questionnaire will also assess parental satisfaction with their child’s oral health and dental esthetics. In the follow-up questionnaire, parents will be asked to report posttreatment complications of the assigned treatment, such as pain from the treated teeth and gingival irritation around the treated teeth.

### Random Allocation, Concealment, and Blinding

With regard to treatment group allocation and intervention, at baseline, participant children were stratified into the following 2 groups: (1) with dmfs score 0 and (2) with dmfs score >0. The children were allocated by a stratified randomization method with varying random block sizes, using a personal computer, into 1 of 3 intervention groups. The unit of treatment allocation was at the subject level. The allocation sequence was generated by a technician who was not involved in the examination and random allocation.

Regarding allocation concealment, a randomization scheme was produced, and a random code for each participant was placed in an opaque envelope. The dental team did not know the treatment allocation before the time of applying the material. The envelope was opened by a dental assistant, and then, the materials were prepared following the assigned intervention of the child according to group allocation. The children and their parents have not been informed about their group allocation. The examiner will also be blinded to the assigned intervention. However, the dentists who will provide the interventions will not be blinded because the agents (SDF, NaF, and placebo) look dissimilar in nature.

### Interventions

Occlusal molar surfaces without cavitated dentine caries lesions were randomly allocated to receive SDF, NaF varnish, or a placebo control. Clinical examinations will be conducted every 6 months after the intervention.

The treatment interventions are as follows: (1) Group A, semiannual topical application of 38% SDF solution (Saforide, Toyo Seiyaku Kasei Co, Ltd); (2) Group B, semiannual topical application of 5% NaF varnish (Duraphat, Colgate Palmolive); (3) Group C, semiannual topical application of a placebo control (tonic water).

The application procedure is as follows:

1. Remove food debris and dental plaque, if any, from the occlusal surface to allow good contact between the study agent and tooth surface.
2. Isolate the occlusal surface of sound primary molars with cotton roll or gauze. For Group A, dispense 1 drop (0.05 mL) of 38% SDF solution, which contains approximately 2.2 mg fluoride ions, in a plastic dappen dish. For Group B, dispense 1 drop (0.25 mL) of 5% NaF varnish, which contains approximately 5.6 mg fluoride ions, in a plastic dappen dish. For Group C, dispense one drop (approximately 0.05 mL) of tonic water in a plastic dappen dish.
3. Slightly bend a microapplicator, and dip it in the agent.
4. Apply the agent with the microapplicator directly onto the occlusal surface of sound primary molars in each quadrant. One application is used for 1 quadrant (1 or 2 primary molars). If a child has 8 sound molars, he or she will receive a maximum of 4 applications on that day.
5. Gently rub the occlusal surfaces with the microapplicator while continuing to isolate the treated teeth whenever possible and ensure that the entire occlusal surfaces are wetted by the agent.
6. Minimize contact of the SDF solution with adjacent gingiva or mucosa to avoid potential soft-tissue irritation.

**Figure 1:**
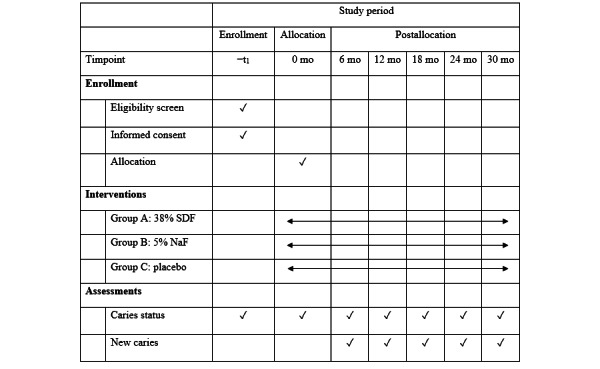
Schedule of subject enrollment, interventions, and outcome assessments. NaF: sodium fluoride; SDF: silver diamine fluoride.

Subsequently, the study children will be instructed not to eat and drink for 30 minutes after application. The intervention will be provided every 6 months. Application of the appropriate agent (either 38% SDF, 5% NaF, or tonic water) will be carried out after an oral examination according to the assigned treatment group. The schedule of subject enrollment, interventions, and outcome assessments is shown in Figure 1.

Parents of all study children will be invited to attend an oral health talk by a dentist once a year. A presentation aided by color photographs and slides about child oral care will be given to parents in the kindergartens. A set of a toothbrush and fluoridated toothpaste will be given to all study children as a souvenir once a year. After the examination, a report on the child’s oral health status, including caries status, and leaflets about the child’s oral health will be sent to the parents.

### Outcome Measure

Information related to the outcomes of the trial will be collected at baseline and thereafter semiannually for 30 months.

#### Primary Outcome 

The treatment will be classified as “success” if an occlusal surface at baseline does not develop cavitated dentin caries lesions at the follow-up examination. “Failure” will be recorded if the surface develops cavitated dentin caries; the tooth has received dental sealant placement, a crown, or occlusal fillings; or the tooth is missing due to caries.

#### Secondary Outcomes

Parent’s satisfaction with their child’s dental health and dental appearance will be measured using a 5-point Likert scale, ranging from 1 (very dissatisfied) to 5 (very satisfied).

Information on the adverse effects of SDF treatment, such as tooth pain, gingival irritation, and systemic toxicity (eg, nausea and vomiting), will be collected through a parental questionnaire within a week after the intervention. During the follow-up examination, the examiner will look for signs and symptoms of potential side effects, including blackening of the treated teeth and adjacent teeth. Regarding parental satisfaction, questionnaires will be distributed to assess parent’s satisfaction with their child’s oral health and dental appearance before the trial and at the 30-month follow-up.

### Sample Size Calculation

Based on previous studies [3,21], the anticipated proportion of sound occlusal surfaces at baseline developing into cavitated dentin lesions at the 30-month follow-up is approximately 25%. An absolute difference of 10% in the rate of new caries development (proportion of occlusal surfaces that develop cavitated dentin lesions at 30 months) between 2 intervention groups is considered to be clinically significant. The estimated sample size is based on the expected proportion of the development of new cavitated occlusal caries in primary molars, with the power of the study set at 90% (β=.1) and with a 2-sided test at the 5% statistical significance level. The sample size per study group, calculated by using the G*Power 3.1.9.2 software (University of Düsseldorf, Germany) is 351 sound occlusal tooth surfaces. The estimated intraclass correlation coefficient (ICC) for dental caries data at the surface level within the individual is approximately 0.5 [22]. Based on our previous survey [3], we anticipated that the mean number of sound occlusal surfaces per child would be 6 at baseline. Following the equation for the required sample size in a multilevel model, the design effect will be 3.5. Thus, the estimated sample size would be at least 1229 sound occlusal surfaces and with at least 205 children in each study group or 615 in total at baseline. The anticipated dropout rate after 30 months is 20%. Thus, 769 children in total need to be recruited at baseline. The estimated participation rate is approximately 90%, and therefore, at least 854 children have to be invited to join the study.

### Data Analysis

The collected data will be entered into a Microsoft Excel file by 2 persons, and the data will be proofread to minimize data entry error. Data will be analyzed using the SPSS software for Microsoft Windows (SPSS Inc). Intraexaminer agreement in caries diagnosis will be assessed by using Cohen kappa statistics. Since more than one molar may be selected from 1 child, multilevel data analysis will be carried out.

A chi-square test and *t* test will be performed, when appropriate, to assess the differences between groups with regard to their baseline demographic background, oral health–related behaviors, and caries experience. A chi-square test will be used to evaluate the differences in new caries development and parental satisfaction among groups. Thus, 2-level generalized estimating equation models with a logit link function (child level and surface level) will be adopted to evaluate the variables influencing the occurrence of occlusal caries at the 30-month follow-up. Independent variables include the child’s background characteristics (gender, place of birth, father’s and mother’s education levels, and family income); the child’s clinical characteristics at baseline (tooth type, such as upper or lower molars and first or second molars, presence of plaque on the occlusal surface, caries experience, and overall oral hygiene); and the child’s oral health–related behaviors (use of fluoride agents, dental visit behavior, and snacking habit). The backward elimination method will be adopted for modeling. A McNemar test will be performed to compare the differences in parent’s satisfaction with their child’s dental appearance and dental health at baseline and the 30-month follow-up. The level of statistical significance for all tests will be set at .05. An intention-to-treat (ITT) analysis will be adopted so that all study children who are randomized and all events will be accounted for in the primary analysis. The relevant government agencies have access to the collected data for the purposes of verifying the integrity of the study data and evaluating compliance with the study protocol. Regarding confidentiality, hard copies of the data will be stored in a sealed box in a locked room during the trial and after publication for 5 years. In addition, the research findings will be presented in scientific conferences for clinicians and health care researchers, and will be disseminated to the community and stakeholders through oral health educational seminars and talks.

## Results

This study was started on September 1, 2020, and the recruitment process ended on September 30, 2021. At present, a total of 791 children are participating in the study. This 30-month clinical trial is expected to be completed in March 2024.

## Discussion

This is a randomized, 3-arm, double-blind, parallel-design clinical trial that aims to investigate the effectiveness of semiannual application of 38% SDF, 5% NaF varnish, and placebo for preventing new caries on occlusal surfaces of primary molars. We hypothesize that SDF has a better effect for preventing occlusal caries than NaF varnish and control owing to its promising results in remineralizing initial enamel caries lesions [23]. The caries-arresting effects of SDF in preschool children have been well documented. However, there is limited information on the effectiveness of SDF for primary prevention purposes in young children. The effect of SDF on caries in primary teeth was systematically reviewed [20,24]. Unfortunately, all included studies in the meta-analysis primarily focused on therapeutic effect, and SDF was applied to decayed teeth. So far, there is insufficient evidence regarding the preventive effect of SDF in primary teeth in vivo. Therefore, it is essential to investigate the caries-preventive effects of SDF versus NaF varnish and placebo control on the occlusal surfaces of the primary molars of preschool children. It should be noted that the children in the placebo group will not receive substandard care, since there are no existing government subsidized programs that provide professionally applied topical fluorides to prevent dental caries in kindergarten children. In fact, all participant children will have additional benefits from regular dental check-ups and receive a free toothpaste and toothbrush. Parents of the study children will receive oral health information through distributed leaflets and oral health seminars in the kindergarten.

Preventing the development of ECC is the ultimate goal of a disease management plan. Based on recent epidemiological studies, ECC remains prevalent globally [25], and the situation is more challenging in low-income countries [26]. An innovative evidence-based preventive program is needed. Since the proposed caries prevention protocol is feasible, affordable, and noninvasive, if shown to be effective, it can be adopted in a school-based or community-based setting to prevent ECC in children from disadvantaged communities. The strength of this study is adequate sample size, thus leading to sufficient study power. A limitation of this study is that the results of this trial conducted in healthy young children may not be translatable to other groups of children who have special health care needs or other age groups. A further study on the preventive effect of SDF conducted in patients with special health care needs is required. We plan to disseminate the research findings to different audiences as follows: (1) For dental practitioners and dental educators, the research findings will be published in international peer-reviewed journals and presented at scientific conferences; (2) For community health care teams, kindergarten teachers, and stakeholders, workshops about caries prevention for preschool children will be arranged in Hong Kong; and (3) For end users (children and parents), a story and brief summary of the research findings will be presented in plain language through newsletters and videos on the faculty website, as well as local multimedia.

The results of this study will provide evidence to strengthen or refute the recommendation regarding the use of SDF for preventing occlusal caries in primary molars. The study findings can guide decision-making among dental practitioners and health policymakers regarding whether SDF should be included in a school-based caries prevention program.

## Appendix

Multimedia Appendix 1 SPIRIT (Standard Protocol Items: Recommendations for Interventional Trials) checklist.

## Abbreviations

dmfs: decayed, missing, and filled tooth surface
ECC: early childhood caries
NaF: sodium fluoride
SDF: silver diamine fluoride
